# Dairy Products and Dairy-Processing Environments as a Reservoir of Antibiotic Resistance and Quorum-Quenching Determinants as Revealed through Functional Metagenomics

**DOI:** 10.1128/mSystems.00723-19

**Published:** 2020-02-18

**Authors:** Elena A. Alexa (Oniciuc), Calum J. Walsh, Laura M. Coughlan, Amal Awad, Cezara A. Simon, Lorena Ruiz, Fiona Crispie, Paul D. Cotter, Avelino Alvarez-Ordóñez

**Affiliations:** a Department of Food Hygiene and Technology, Universidad de León, León, Spain; b Teagasc Food Research Centre, Fermoy, County Cork, Ireland; c Bacteriology, Mycology and Immunology Department, Faculty of Veterinary Medicine, Mansoura University, Mansoura, Egypt; d Dairy Research Institute, Spanish National Research Council, Instituto de Productos Lácteos de Asturias—CSIC, Villaviciosa, Spain; e Institute of Food Science and Technology, Universidad de León, León, Spain; f APC Microbiome Ireland, University College Cork, Cork, Ireland; University of Sao Paulo

**Keywords:** antibiotic resistance, food safety, functional metagenomics, dairy products, quorum quenching

## Abstract

Here, the role of the dairy-processing chain as a reservoir of antimicrobial resistance (AR) determinants and a source of novel biocontrol quorum-sensing inhibitors is assessed through a functional metagenomics approach. A metagenomic library comprising ∼22,000 recombinant clones was built from DNA isolated from raw milk, raw milk cheeses, and cheese-processing environment swab samples. The high-throughput sequencing of 9,216 recombinant clones showed that lactic acid bacteria (LAB) dominated the microbial communities of raw milk cheese, while Gram-negative microorganisms of animal or soil origin dominated the microbiota of raw milk and cheese-processing environments. Although functional screening of the metagenomic library did not recover potential quorum-sensing inhibitors, *in silico* analysis using an in-house database built specifically for this study identified homologues to several genes encoding proteins with predicted quorum-quenching activity, among which, the QsdH hydrolase was the most abundant. *In silico* screening of the library identified LAB, and especially Lactococcus lactis, as a relevant reservoir of AR determinants in cheese. Functional screening of the library allowed the isolation of 13 recombinant clones showing an increased resistance toward ampicillin, which in all cases was accompanied by a reduced susceptibility to a wide range of β-lactam antibiotics. This study shows that the dairy-processing environment is a rich reservoir of AR determinants, which vary by sample source, and suggests that combining next-generation sequencing with functional metagenomics can be of use in overcoming the limitations of both approaches.

**IMPORTANCE** The study shows the potential of functional metagenomics analyses to uncover the diversity of functions in microbial communities prevailing in dairy products and their processing environments, evidencing that lactic acid bacteria (LAB) dominate the cheese microbiota, whereas Gram-negative microorganisms of animal or soil origin dominate the microbiota of milk and cheese-processing environments. The functional and *in silico* screening of the library allowed the identification of LAB, and especially Lactococcus lactis, as a relevant reservoir of antimicrobial resistance (AR) determinants in cheese. Quorum-quenching (QQ) determinants were not recovered through the execution of wet-lab function-based screenings but were detected through *in silico* sequencing-based analyses.

## Abstract (precis)

The study shows the potential of functional metagenomics analyses to uncover the diversity of functions in microbial communities prevailing in dairy products and their processing environments, evidencing that lactic acid bacteria (LAB) dominate the cheese microbiota, whereas Gram-negative microorganisms of animal or soil origin dominate the microbiota of milk and cheese-processing environments. The functional and *in silico* screening of the library allowed the identification of LAB, and especially Lactococcus lactis, as a relevant reservoir of antimicrobial resistance (AR) determinants in cheese. Quorum-quenching (QQ) determinants were not recovered through the execution of wet-lab function-based screenings but were detected through *in silico* sequencing-based analyses.

## INTRODUCTION

Cheeses made from unpasteurized milk are more attractive to some consumers due to the development of more diverse flavor and aroma profiles than those of cheeses manufactured with pasteurized milk. However, the coexistence of an expanded microbial richness during the manufacture of raw milk cheeses may also influence the microbial safety of the product (1). This influence may be either detrimental, through the presence of pathogens, or beneficial, through the presence of saprophytic microorganisms which may prevent the overgrowth of pathogens.

Lactic acid bacteria (LAB) are the dominant bacteria in fermented dairy foods, including cheeses, and are known to positively influence the flavor, aroma, or texture of the end product (1). In addition, during cheese manufacturing and especially during ripening, the cheese surface is exposed to an unsterile environment where a wide range of LAB and other opportunistic microorganisms, including some undesirable taxa such as staphylococci, Listeria monocytogenes, Escherichia coli, and others, can become established (1, 2). Ultimately, foodborne fermentation-associated bacteria and other associated microorganisms occurring in cheese can originate from the microbiota naturally present in raw milk, can be selected commercial starters that are specifically added, or can be transferred to the product from the processing environment (1).

Historically, complex microbial communities, including those occurring in fermented foods and food-processing environments, have been difficult to characterize due to the fact that classic culture-dependent approaches cannot recover all microorganisms present in a population, with particular issues relating to recovering difficult-to-culture or viable but not culturable microorganisms (3). With the advent of culture-independent molecular tools, metagenomics approaches have proved to be particularly valuable when overcoming such limitations. One such metagenomics-based approach is functional metagenomics. Functional metagenomics analyses can be carried out through the isolation and purification of DNA coming from an environmental sample, its cloning into an expression vector (e.g., a plasmid or fosmid), its expression in a suitable host (usually E. coli), and the characterization of the recombinant clones via sequencing- or phenotype-based approaches or both (4). The creation of active recombinant clones depends upon the successful expression of genes from metagenomic DNA and secretion of the functional protein by the host cell and can be identified based on the detection of an activity of interest (4). Thus, for example, functional metagenomics approaches were previously used to identify novel enzymes or enzymatic pathways (5–7) or to characterize determinants of resistance to antimicrobials (antimicrobial resistance [AR]) (8, 9) or utilization of heavy metals (10).

Research carried out in the last decade points toward the possibility of the transmission of antimicrobial resistance (AR) via the food chain and toward the relevance of food-processing environments as potential hot spots for the emergence and spread of AR (11). Some previous studies have assessed the distribution patterns of AR determinants in dairy-derived products and associated processing environments using functional metagenomics methodologies (9, 12, 13). As an example, DeVirgiliis et al. (12) showed that various fosmid-borne LAB-derived genes produce an AR phenotype in an E. coli host by screening a metagenomic library constructed from samples of mozzarella di Bufala Campana Italian cheese type. Although they described a low recovery of resistant recombinant clones, corresponding to a low occurrence of antibiotic-resistant bacteria, this study showed that functional metagenomics methodologies are sensitive and efficient for identifying AR determinants and can overcome the limitations of culture-dependent methods (3).

Strategies focused on inhibiting cell-to-cell communication or quorum sensing (QS) are among the most promising approaches for combatting antimicrobial-resistant microorganisms. All QS systems utilize small secreted signaling molecules known as autoinducers. These include acylated homoserine lactones or autoinducer-1 (used by Gram-negative bacteria), peptide signals (used by Gram-positive bacteria), and autoinducer-2 (used by both Gram-negative and Gram-positive bacteria) (14). Functional metagenomics approaches have also been explored as a way to identify enzymatic activities capable of inhibiting QS, also known as quorum-quenching (QQ) determinants (15, 16), commonly through the degradation of autoinducer molecules. These novel QQ determinants have been proposed as novel tools for controlling antimicrobial-resistant microorganisms through inhibiting the full expression of their virulence potential (17, 18).

The objectives of this study were (i) to construct a fosmid metagenomic library containing total DNA extracted from the microbiota of raw cow milk, artisanal raw milk cheeses, and dairy-related processing environments, (ii) to characterize the inserts of the metagenomic library through high-throughput sequencing, and (iii) to undertake function-based and *in silico* sequencing-based screenings of the metagenomic library in search of AR determinants conferring resistance to antimicrobials belonging to different pharmacological classes and QQ determinants capable of inhibiting autoinducer-1 or autoinducer-2 QS molecules.

## RESULTS

### Metagenomic library construction and assessment of its microbial diversity.

To maximize the representation of the entire cheese microbiota and obtain genomic DNA representing a large proportion of the microbial taxa potentially entering the human gastrointestinal tract through cheese consumption, pools of samples coming from different compartments of the cheese-processing chain (i.e., raw milk from a dairy farm, swab samples from processing environments of four cheese-producing facilities, and four samples of different raw milk cheeses bought from retailers) were used to build a library of ∼22,000 recombinant clones (∼850 Mb of DNA), comprising ∼185 Mb of DNA from raw milk, ∼480 Mb of DNA from raw milk cheeses, and ∼185 Mb of DNA from food-processing environments. Considering that the library contains clones with 40-kb-average inserts and assuming an average genome size of 4 Mb for bacteria, the constructed metagenomic library would contain a DNA quantity equivalent to more than 200 bacterial genomes. While noting that not all clone inserts will be different, this library size nonetheless provides a reasonable opportunity to detect specific sequences of interest in the genomes of microorganisms with more than 1% relative abundance in the total metagenome of the samples of origin.

The method used for library construction involves the cloning of randomly sheared end-repaired DNA, leading to the generation of highly random DNA fragments. To confirm this, three different approaches were followed. First, a random selection of 30 recombinant clones (10 from raw milk-recovered DNA, 10 from cheese-recovered DNA, 10 from processing environment-recovered DNA) were characterized through restriction analysis with the enzyme ApaI, which evidenced the presence of diverse restriction profiles encompassing different numbers and sizes of DNA fragments (data not shown). Second, the species of origin of the inserted DNA was identified for this random selection of 30 recombinant clones through partial Sanger sequencing of the insert-flanking regions using specific primers targeting the pCC1FOS vector. These analyses showed that (i) raw milk-associated clones may contain a significant amount of eukaryotic DNA, likely coming from milk somatic cells, (ii) cheese-associated clones mainly contained DNA from Lactococcus lactis strains, likely used as the starter cultures during manufacturing, and (iii) clones associated with processing environments are more heterogeneous and contained DNA from a diverse set of microorganisms, such as *Lactococcus* spp., *Psychrobacter* spp., *Stenotrophomonas* spp., *Klebsiella* spp., or *Moritella* spp. The third approach used for assessing the microbial diversity within the metagenomics library involved the characterization through high-throughput sequencing of pools of randomly selected clones from different categories (pools of raw milk-associated clones, pools of processing environment-derived clones, pools of cheese-associated clones derived from two different batches of cheese DNA, and pools containing both raw milk- and processing environment-associated clones), including 9,216 recombinant clones in total.

**FIG 1:**
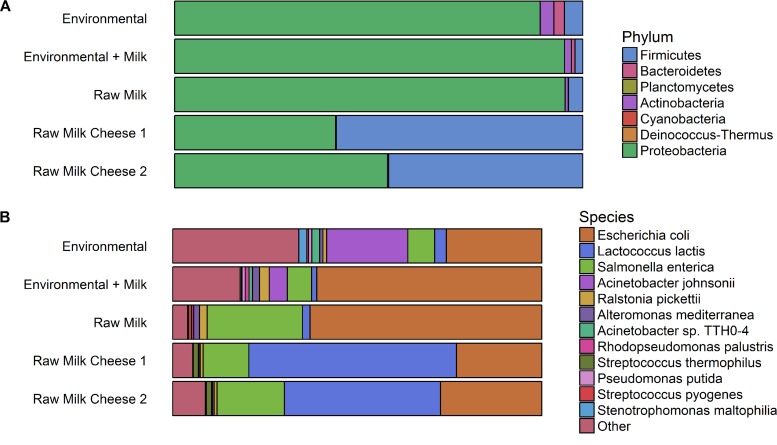
Taxonomic profiles of metagenomic libraries obtained from sampled sources following whole-metagenome shotgun sequencing and removal of reads from host and vector DNA. Stacked bar charts represent proportions of metagenomic reads which could be classified by Kraken2 at phylum (A) and species (B) levels.

Taxonomic assignments obtained from the metagenomic library sequencing, after removal of reads from host and vector DNA, showed differences in microbial richness and composition between samples of fresh raw milk, artisanal raw milk cheeses, and processing environmental samples, both at phylum and at species levels (Fig. 1A and B). Although *Proteobacteria* and *Firmicutes* were the most abundant phyla in all reservoirs, followed by *Actinobacteria* and *Bacteroidetes*, *Firmicutes* was a dominant phylum in cheese-associated clones, while in raw milk and cheese-processing environments, *Proteobacteria* were clearly more abundant than *Firmicutes* (Fig. 1A).

At the species level, it was confirmed that the two batches of DNA from artisanal raw milk cheeses were both dominated by L. lactis, with relatively high abundances of E. coli and Salmonella enterica subsp. *enterica*, and contained many other LAB belonging to the *Lactococcus* and *Streptococcus* genera at a lower frequency. E. coli was the most prevalent species in raw milk and cheese-processing environments, followed by Acinetobacter johnsonii and S. enterica subsp. *enterica* in processing environments and raw milk, respectively (Fig. 1B). Overall, environmental samples harbored the highest diversity of species, while raw milk harbored the lowest (see Fig. S1 and Table S3 in the supplemental material). The former was characterized by a large number of low-abundance species, such as Stenotrophomonas maltophilia, Psychrobacter alimentarius, and *Pseudomonas* spp., among others. More information on variability in microbial composition among different pools of recombinant clones from different origins is provided in Fig. S2 and S3.

10.1128/mSystems.00723-19.1 FIG S1 Comparison of within-sample taxonomic diversity between sample sources as measured by Simpson and Shannon indices at phylum (A) and species (B) levels. Lines joining sources denote statistically significant differences. A full list of associated *P* values is provided in Table S3. Download FIG S1, EPS file, 2.1 MB. Copyright © 2020 Alexa (Oniciuc) et al. 2020 Alexa (Oniciuc) et al. This content is distributed under the terms of the Creative Commons Attribution 4.0 International license.

10.1128/mSystems.00723-19.2 FIG S2 Taxonomic profile of metagenomic libraries obtained from samples following whole-metagenome shotgun sequencing and removal of reads from host and vector DNA. Stacked bar charts represent proportions of metagenomic reads which could be classified by Kraken2 at the phylum level. Download FIG S2, EPS file, 0.1 MB. Copyright © 2020 Alexa (Oniciuc) et al. 2020 Alexa (Oniciuc) et al. This content is distributed under the terms of the Creative Commons Attribution 4.0 International license.

10.1128/mSystems.00723-19.3 FIG S3 Taxonomic profile of metagenomic libraries obtained from samples following whole-metagenome shotgun sequencing and removal of reads from host and vector DNA. Stacked bar charts represent proportions of metagenomic reads which could be classified by Kraken2 at the species level. Download FIG S3, EPS file, 0.1 MB. Copyright © 2020 Alexa (Oniciuc) et al. 2020 Alexa (Oniciuc) et al. This content is distributed under the terms of the Creative Commons Attribution 4.0 International license.

### Identification of QQ determinants.

**FIG 2:**
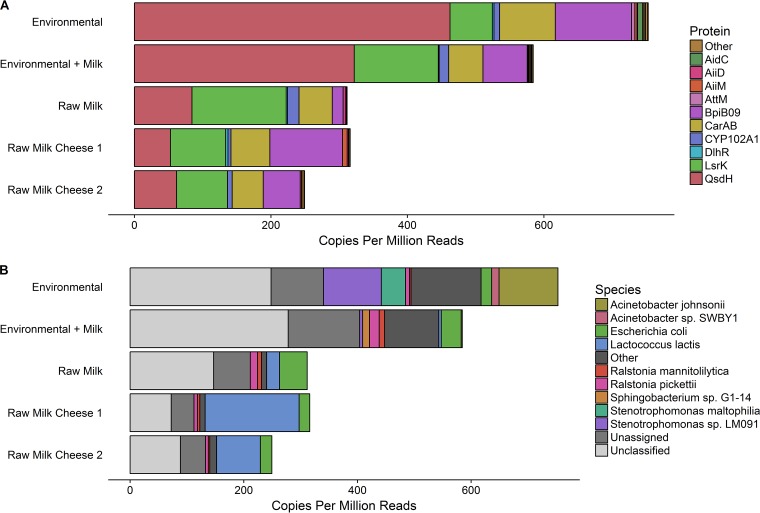
Quorum-quenching determinants identified by whole-metagenome shotgun sequencing of metagenomic libraries. (A) Quorum-quenching genes identified, normalized as copies per million paired-end reads per sample source. The 10 most frequently identified genes are shown, and the remaining genes are grouped together and labeled “other.” (B) Species-level classification of QQ determinants normalized as copies per million paired-end reads per sample source. Sequences which could not be classified by Kraken2 are labeled unclassified while sequences which could not be classified to a species are labeled unassigned.

The functional screening of the metagenomic library to identify determinants of quorum-quenching activity showed the absence of colorless or lightless halos in the Chromobacterium violaceum DSMZ 30191 and Vibrio harveyi DSMZ 19623 bioassays, which revealed that no recombinant clones were able to degrade the autoinducer-1 or autoinducer-2 molecules produced by these two indicator strains. Nevertheless, the *in silico* screening of the inserts of the 9,216 recombinant clones characterized through high-throughput sequencing, using an in-house database specifically built in this study to search for known quorum-quenching determinants, was able to identify 2,923 homologues to several genes encoding proteins with predicted QQ activity, among which, the QsdH hydrolase, with predicted autoinducer-1 inhibitory activity, was the most abundant, particularly in the food-processing environment, followed by the LrsK kinase, a predicted autoinducer-2 inhibitor. Other less abundant QQ determinants included CarAB (a carbamoyl phosphate synthase), BpiB09 (an oxidoreductase), and other proteins in lower proportions (e.g., CYP102A1, AiiM, DlhR, and AiiD) (Fig. 2A). Sixty-two percent of reads predicted to encode QQ determinants were assigned to particular taxa by Kraken2. Of these, 72.7% were classified to the species level. The species to which the QQ determinants were most commonly assigned was L. lactis, followed by E. coli, *Stenotrophomonas* sp., and, particularly in environmental samples, *A. johnsonii* (Fig. 2B). Of particular interest was the case of L. lactis, with a high abundance of QQ-regulated proteins being found assigned to this species in cheese samples, while they were less represented in raw milk samples and completely absent in samples from processing environments.

### Identification of antibiotic resistance determinants.

Two different approaches were followed to identify antibiotic resistance determinants harbored by the inserts of the constructed metagenomics library.

**FIG 3:**
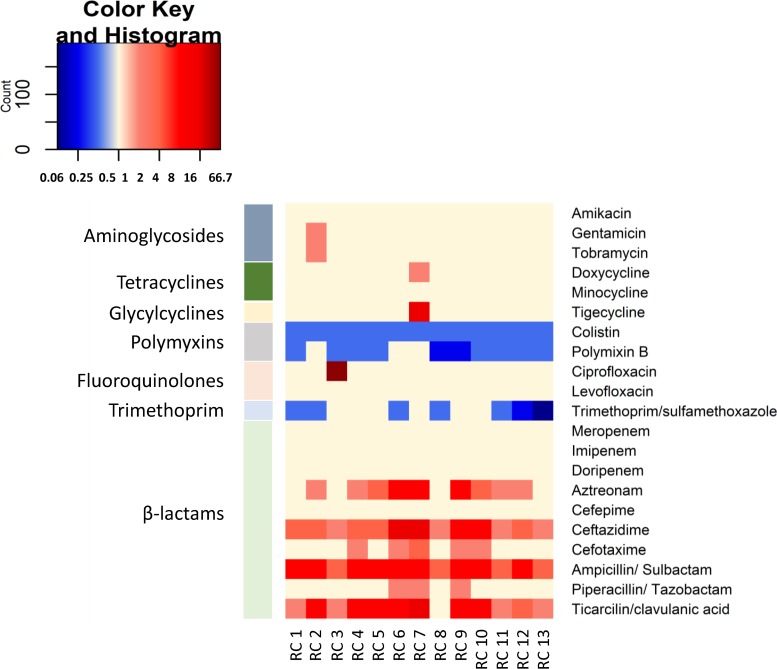
Fold change in susceptibility to the antimicrobials included in the generic Sensititre panel GNX3F- for nonfastidious Gram-negative bacteria of the 13 recombinant clones showing an increased resistance toward ampicillin compared to that of the E. coli host strain. Increases in resistance are shown in red, while increases in susceptibility are shown in blue.

First, the metagenomic library was functionally screened on agar medium supplemented with the antibiotic ampicillin, at its MIC for the E. coli EPI300-T1^R^ host strain (16 mg/liter), to identify determinants of resistance to β-lactam antibiotics. Functional screening by replica plating of the ∼22,000 library clones on LB agar plates containing ampicillin led to the selection of 13 resistant clones. Secondary screening of the resulting resistant colonies performed on freshly obtained LB agar plates containing 16 mg/liter of ampicillin confirmed that all resistant clones were able to grow in the presence of this antibiotic at its MIC for the E. coli host strain, while the E. coli host strain was unable to do so. When the susceptibility of the 13 recombinant clones to a range of antibiotics in comparison to that of the E. coli EPI300-T1^R^ host strain was assessed using Sensititre panels in order to identify multidrug resistance profiles, it was observed that most ampicillin-resistant recombinant clones showed also a decreased susceptibility to a wide range of other β-lactam antibiotics, such as aztreonam, ceftazidime, cefotaxime, ampicillin-sulbactam, piperacillin-tazobactam, and ticarcillin-clavulanic acid, with MICs from 2- to 16-fold higher than those obtained for the E. coli host strain. In addition, one clone (RC3) showed a sharp increase in resistance to the fluoroquinolone ciprofloxacin, one clone (RC2) showed a slight decrease in susceptibility to aminoglycosides (gentamicin and tobramycin), and one clone (RC7) showed increased resistance to the tetracycline doxycycline and the glycylcycline tigecycline. Finally, several recombinant clones showed a slightly increased susceptibility to the polypeptide antibiotics colistin and polymyxin B and to trimethoprim-sulfamethoxazole (Fig. 3).

To further characterize the genomic features of the AR clones, fosmid DNA was extracted from colonies showing an increased resistance to ampicillin and subjected to restriction analysis using ApaI, which fragments the pCC1FOS vector at a unique site. All the ampicillin-resistant clones displayed different patterns with almost no overlapping bands, with differences in the number and size of bands suggesting the occurrence of different inserts, probably generated from distinct genomes within the metagenomic DNA (data not shown). The characterization of the 13 fosmids through shotgun sequencing showed that the insert size ranged between 18 and 40 kb. Most inserts were assigned to Streptococcus salivarius subsp. *thermophilus* genomic sequences, L. lactis genomic sequences, or both, and interestingly, one resistant clone, RC11, could not be assigned to any known microbial species (Table 1). The mapping of the sequencing data to the CARD database to identify known AR determinants revealed the presence of different genes previously identified as conveying resistance to tetracyclines (*tetA*, *tetB*, or *tetO*), glycopeptides (*vanRM*, *vanUG*, *vanXYC*, *vanYB*, and *vanTC*), β-lactams (*pbp2X*), macrolides (*msrC* and *carA*), and streptogramins (*vgaALC* and *vgaA*). Additionally, the thorough inspection of the annotated contigs allowed the identification of several genes putatively encoding enzymes involved in peptidoglycan biosynthesis and/or modification. These included *N*-acetylmuramoyl-l-amidases, a phospho-*N*-acetylmuramoyl-pentapeptidase-transferase, a d-alanine ligase, and several other peptidoglycan modification enzymes (e.g., phosphoserine phosphatase, racemase, and ligase). Interestingly, the insert of one of the clones (RC8) harbored a homologue to the two-component regulatory system LiaS/LiaR, which was previously shown to be involved in coordinating resistance to various cell wall-active antibiotics (19). Moreover, in some clones showing an increased resistance to ribosome-targeting antibiotics, such as aminoglycosides, it was possible to identify genes putatively encoding an RNA methyltransferase, previously described as having a role in resistance to this antibiotic family (20), and a serine acetyltransferase, which could be involved in the enzymatic modification of aminoglycosides. Finally, some fosmids contained genes encoding potential efflux pumps, and all fosmids contained open reading frames (ORFs) annotated as encoding hypothetical proteins.

**TABLE 1:** Drug resistance profile and insert sequencing results of ampicillin-resistant recombinant clones

Recombinant clone	Resistant phenotype	Insert length (bp)	Predicted species	Putative relevant gene for AR^*a*^	Predicted mechanism	Other relevant ORFs for AR
RC1	Aminoglycosides, β-lactams	18,836	S. thermophilus			*N*-Acetylmuramoyl-l-alanine amidase, ligase, RNA methyltransferase
RC2	Aminoglycosides, β-lactams	18,467	S. thermophilus			*N*-Acetylmuramoyl-l-alanine amidase, ligase, serine acetyltransferase
RC3	Fluoroquinolones, β-lactams	24,142	L. lactis	*arnA*	Bifunctional polymyxin resistance	Formyltransferase, Tn*916*, stress response regulator *gls24*
RC4	β-Lactams	30,119	S. thermophilus	*poxtA*, *msrC*, *tetA*, *tetB*	Ribosomal protection and efflux pumps	d-Alanine poly(phosphoribitol) ligase subunit 2, RNA methyltransferase
RC5	β-Lactams	40,868	S. thermophilus	*vanRM*, *evgA*, *vgaA*	Antibiotic target alteration, efflux pumps, ribosomal protection	Peptidase SppA
RC6	β-Lactams	36,330	S. thermophilus	*mel*, *carA*, *pbp2X*	Ribosomal protection, PBP^*b*^ mutations	Phospho-*N*-acetylmuramoyl-pentapeptidase transferase, RNA methyltransferase, *N*-acetyltransferase YvbK
RC7	Tetracyclines, β-lactams	22,341	S. thermophilus			
RC8	β-Lactams	29,850	S. thermophilus, L. lactis	*tetO*, *goIS*, *sdiA*, *tetA*	Ribosomal protection and efflux pumps	LiaS/LiaR, ligase, glyceraldehyde 3-phosphate dehydrogenase
RC9	β-Lactams	31,279	S. thermophilus	*tsnR*, *vanYB*	Antibiotic target alteration	*N*-Acetylmuramoyl-l-alanine amidase, exo-glucosaminidase LytG, ligase, RNA methyltransferase
RC10	β-Lactams	24,406	S. thermophilus	*vanXYC*, *tsnR*	Antibiotic target alteration	D-alanyl-D-alanine carboxypeptidase, exo-glucosaminidase LytG, ligase, RNA methyltransferase, serine acetyltransferase
RC11	β-Lactams	35,242	No assignation			
RC12	β-Lactams	43,395	S. thermophilus	*bcrA*, *vgaALC*, *vanTC*, *vanUG*	Antibiotic target alteration, efflux pumps, ribosomal protection	Racemase, ligase, phosphoserine phosphatase, 23S RNA methyltransferase
RC13	β-Lactams	21,146	E. coli			Cysteine peptidase, peptidase SppA

a AR, antibiotic resistance.

b PBP, penicillin-binding protein.

**FIG 4:**
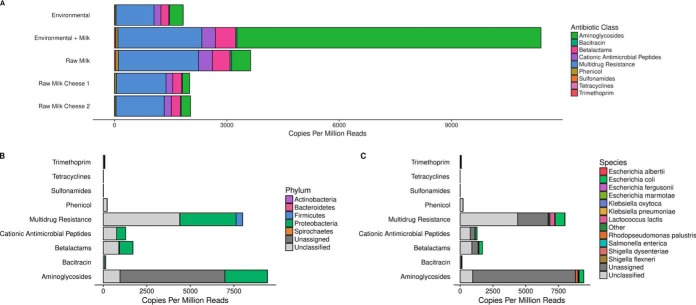
Antibiotic resistance determinants identified by whole-metagenome shotgun sequencing of metagenomic libraries. (A) Classes of antimicrobials to which resistance determinants were identified, normalized as copies per million paired-end reads per sample source. (B) Phylum-level classification of AR determinants normalized as copies per million paired-end reads per class. Reads which could not be classified by Kraken2 are labeled unclassified, while reads which could not be classified to a phylum are labeled unassigned. (C) Species-level classification of AR determinants normalized as copies per million paired-end reads per class. Reads which could not be classified by Kraken2 are labeled unclassified, while reads which could not be classified to a species are labeled unassigned.

The second approach involved the *in silico* screening of the inserts of the 9,216 recombinant clones characterized through high-throughput sequencing in the search for known antibiotic resistance determinants. The identified resistome profiles were broadly similar among raw milk, raw milk cheeses, and environmental swabs, with the most abundant AR determinants being those assigned to the “multidrug resistance” category, followed by determinants of resistance to aminoglycosides, beta-lactams, and cationic antimicrobial peptides (Fig. 4A), while the pool of samples containing environmental and milk DNA were dominated by genes predicted to confer resistance to aminoglycosides, particularly the aph(3′) aminoglycoside *O*-phosphotransferase (Fig. 4A; see also Fig. S4).

10.1128/mSystems.00723-19.4 FIG S4 Antimicrobial resistance genes identified by whole-metagenome shotgun sequencing of metagenomic libraries. Data are normalized as copies per million paired-end reads per sample source. Download FIG S4, EPS file, 0.1 MB. Copyright © 2020 Alexa (Oniciuc) et al. 2020 Alexa (Oniciuc) et al. This content is distributed under the terms of the Creative Commons Attribution 4.0 International license.

**FIG 5:**
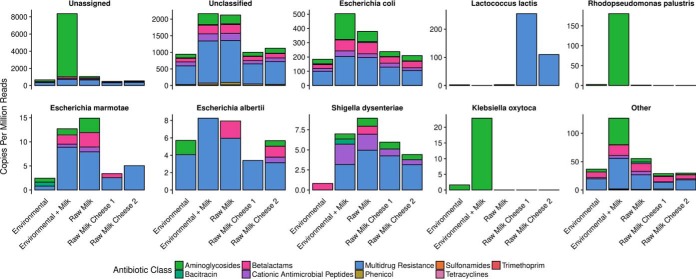
Classes of antimicrobials to which resistance determinants were identified in the most frequently detected species. Reads which could not be classified by Kraken2 are labeled unclassified, while reads which could not be classified to a species are labeled unassigned. The 7 most frequently identified species are shown, and all remaining species are grouped together as “other.” Data are normalized as copies per million paired-end reads per sample source.

Only 35% of the identified resistance determinants were able to be taxonomically assigned at the phylum level, the most abundant originating from *Proteobacteria*, followed by a smaller proportion from *Firmicutes* (Fig. 4B). Only 11% of identified resistance determinants were able to be taxonomically assigned to the species level, with the most commonly identified originating from E. coli and L. lactis (Fig. 4C). The resistome profiles were broadly similar across sample sources, with the exception of high levels of apparently multidrug-resistant L. lactis in raw milk cheese samples and aminoglycoside-resistant Rhodopseudomonas palustris and Klebsiella oxytoca, associated almost entirely with a batch of samples containing environmental and milk DNA. The latter niche was characterized by large quantities of aminoglycoside resistance genes, of which the vast majority were not able to be taxonomically classified at the species level (Fig. 5). More information on variability in the resistomes among different pools of recombinant clones from different origins is provided in Fig. S5.

10.1128/mSystems.00723-19.5 FIG S5 Classes of antibiotics to which resistance determinants were identified by whole-metagenome shotgun sequencing of metagenomic libraries. Data are normalized as copies per million paired-end reads per sample. Download FIG S5, EPS file, 0.1 MB. Copyright © 2020 Alexa (Oniciuc) et al. 2020 Alexa (Oniciuc) et al. This content is distributed under the terms of the Creative Commons Attribution 4.0 International license.

## DISCUSSION

The library of ∼22,000 recombinant clones obtained in this study from dairy products and their associated microenvironments encompassed a high diversity of DNA inserts from different microbial taxa, with taxonomic assignments depending on the sample type. *Firmicutes* was a dominant phylum, and L. lactis the dominant species, in cheese-associated clones. The high representation of sequences assigned to L. lactis, and in lower proportions to other *Lactococcus* spp. and *Streptococcus* spp., found in raw milk cheeses, agrees with the findings by other researchers (21–24), whereas Stellato et al. (25) demonstrated the coabundance of both LAB and spoilage-associated bacteria in mozzarella, ricotta, and associated environmental samples. LAB occurring in raw milk cheeses may belong to the starter cultures used during manufacturing or could originate from the raw materials or the processing environments. Clones obtained from raw milk and food-processing environments contained a minor proportion of sequences assigned to LAB accompanied by a high proportion of sequences assigned to Gram-negative bacteria. In addition, in processing environments, a more diverse range of microbial species was identified, and sequences were frequently assigned to E. coli and Acinetobacter spp. Studies based on culture-independent methodologies have previously shown that hundreds of different microbial species can be present in a single processing facility, but only a few taxa of residential bacteria commonly dominate processing environments, as reviewed by Møretrø and Langsrud (26), among which *Enterobacteriaceae* and Acinetobacter spp. are frequently identified.

In contrast to direct shotgun sequencing of total metagenomic DNA, which only identifies determinants showing homology to others previously described and included in the available databases, functional metagenomics can help identify novel determinants providing a given functionality to the host cell, detected through the execution of lab-based screenings. Here, lab-based screenings were complemented with shotgun sequencing analyses of a large collection of recombinant clones. Even though direct shotgun sequencing of total metagenomic DNA would have resulted in a larger sample coverage than shotgun sequencing of the library recombinant clones, the combined approach followed in this study allowed us to identify carriage of already known AR and QQ determinants, even if those activities were not recovered in some of the function-based screenings executed. Indeed, while *in silico* sequencing-based screenings allowed the identification of DNA fragments showing a high homology to known AR and QQ genes, most of them could not be recovered during the execution of wet-lab function-based screenings. This can be due to a lack of expression of DNA fragments coming from a wide diversity of microbial taxa in a relatively domesticated host such as E. coli, defects during posttranslational modification of proteins in the host strain, the possibility of DNA fragments being too short to contain fully operational gene cluster or operons (4, 27), or limitations associated with the functional assays.

Regarding the AR screenings, it is worth highlighting that both sequencing-based and function-based metagenomics approaches can uncover determinants involved in intrinsic antimicrobial resistance or in acquired antimicrobial resistance, with the latter being especially relevant for food safety given their ability to be horizontally transmitted. Here, interestingly, while the *in silico* analyses were not always capable of assigning the identified AR determinants to particular microbial taxa, those were mostly assigned to E. coli and, in smaller proportions, to other members of the *Proteobacteria*. Moreover, the profiles of AR determinants assigned to most microbial taxa during the *in silico* analyses were very similar, with the exception of increased multidrug-resistant L. lactis in raw milk cheeses and higher levels of aminoglycoside-resistant *Proteobacteria* in the processing environment. Therefore, raw milk cheese samples appeared to harbor a large proportion of multidrug-resistant L. lactis, supporting the results of the functional screening of the library on agar plates supplemented with ampicillin, which only recovered clones harboring DNA inserts coming from raw milk cheese samples and primarily contained DNA from LAB, and particularly from Streptococcus thermophilus and L. lactis, presumably due to their role as starter cultures. The multidrug resistance detected in L. lactis was conferred exclusively by the MsbA efflux protein, which was previously shown to confer resistance to multiple antimicrobials, including erythromycin (28), against which L. lactis is not intrinsically resistant (29). This gene was not detected in any other species in this study. Previous studies using functional metagenomics approaches to assess the microbial communities of cheese have also reported the detection of AR determinants linked to LAB. In a study using a functional metagenomics analysis to study mozzarella di Bufala Campana cheese (12), clones containing homologues to the *ampR* and *kanR* genes, conferring resistance to ampicillin and kanamycin, respectively, were recovered. The authors concluded that, most likely, S. thermophilus and Lactobacillus helveticus were the carriers of such AR determinants. In another study evaluating a metagenomic library built using DNA isolated from a raw milk blue-veined cheese in Spain, several tetracycline resistance determinants (*tetA*, *tetL*, *tetM*, and *tetS*) were identified, most of them being conveyed in DNA fragments showing high homology to plasmids from Gram-positive and Gram-negative bacteria, such as plasmids pMAK2 and pO26-Vir from S. enterica subsp. *enterica* and E. coli. In addition, these authors showed that various tetracycline resistance determinants were embedded or in proximity to sequences homologous to those of LAB-derived species (9). It is reasonable to believe that members of LAB isolated from dairy products can act as reservoirs for different AR genes, as was previously reported (9, 12, 28–30). In our study, the use of a combined approach involving both function-based and *in silico* sequencing-based analyses to characterize a large metagenomics library built with DNA isolated from different compartments of the cheese production chain allowed us to identify L. lactis as a major reservoir and source of AR determinants within the dairy production chain. Moreover, the recovery of one particular resistant clone, RC11, was notable in that the insert DNA could not be assigned to any known microbial taxa, showing yet again that functional metagenomics approaches can be useful to identify novel uncovered functions.

The sequencing of the fosmids recovered from the resistant clones capable of growing on ampicillin-supplemented medium revealed the presence of sequences with high homology to genes previously described as determinants of resistance to different antimicrobial classes, such as tetracyclines, glycopeptides, β-lactams, macrolides, and streptogramins. However, phenotypic tests with strains containing these fosmids showed a consistent decrease in susceptibility to a wide range of β-lactam antibiotics, accompanied in the case of only three clones (RC2, RC3, and RC7) by an increase in resistance to some antibiotics belonging to other classes, such as fluoroquinolones, aminoglycosides, or tetracyclines. This fact shows that the resistance determinants harbored in the DNA inserts conveyed a general protection to the host cell against β-lactam molecules and confirms that not all ORFs predicted to act as resistance determinants are phenotypically expressed in the metagenomic library clones. In the recombinant clone RC6, the increased resistance to β-lactam antibiotics could be due to the presence of a gene encoding a penicillin binding protein (PBP). Several PBPs involved in cell wall biosynthesis have been described in resistant strains, including the PBP2a described in Staphylococcus aureus or PBP2x in Streptococcus pneumoniae, respectively, that have low affinity for β-lactams (31, 32). In the recombinant clone RC8, the phenotype could be due to the presence of sequences showing homology to the two-component regulatory system LiaS/LiaR, which was previously shown to be involved in resistance to various cell wall-active antibiotics (19). For the rest of the resistant clones, the decreased susceptibility to β-lactams could be due to the presence of various genes encoding enzymes involved in peptidoglycan biosynthesis and/or modification pathways, such as *N*-acetylmuramoyl-l-amidases, a phospho-*N*-acetylmuramoyl-pentapeptidase-transferase, a d-alanine ligase, and several other peptidoglycan modification enzymes, to the presence of genes putatively encoding nonspecific efflux pumps (19), or to some hypothetical proteins of as-yet-unknown function.

### Conclusion.

This study shows the potential of functional metagenomics analyses to uncover the diversity of functions in complex microbial populations, such as those of milk, artisanal milk chesses, and related processing environments. The study of a metagenomic library containing ∼22,000 clones evidenced that LAB dominate the microbial communities of cheese, while Gram-negative microorganisms of animal or soil origin dominate the microbiota of milk- and cheese-processing environments. The functional and *in silico* screening of the library allowed the identification of LAB, and especially L. lactis, as a relevant reservoir of AR determinants in cheese. However, the low frequency of resistant clones recovered from the metagenomic library may likely correspond to a generally low occurrence of antibiotic-resistant bacteria in the food product. The fact that *in silico* analyses identified putative AR and QQ determinants that could not be recovered through functional screenings shows the current limitations of functional metagenomics approaches, related to the lack of sufficient expression of the genes, and also highlights the value of supporting and complementary sequencing-based approaches in spite of the intrinsic limitation of lower sample coverage as a result of high levels of unnecessary sequencing of vector and eukaryotic DNA.

## MATERIALS AND METHODS

### Sampling of raw milk, artisanal raw milk cheeses, and dairy-relevant environmental reservoirs.

To increase the representativeness of the cheese production chain, the sampling targeted the collection of a wide range of samples from different points of the production and distribution chain and from different processing facilities rather than the collection of a large number of samples from a particular reservoir or a particular processing facility. Taking this into account, from 2014 to 2015, two samples of fresh raw cow milk from a local farm, four samples of artisanal raw milk cheeses bought from retailers (three of them made from cow’s milk and one from goat’s milk), and 160 swab samples from processing environments of four cheese production businesses (40 swab samples from each food business) located in Southern and Western Ireland were collected. Raw milk and raw milk cheese samples, as well as dairy-processing plants producing raw milk dairy products, were exclusively selected in order to access the more diverse milk- and dairy-associated microbiota profiles associated with these niches. Sampled processing environments included drains, sinks, floors, production equipment, and work surfaces, with representation of both food-contact and non-food-contact environments. A more detailed description of the samples used to construct the metagenomic library is shown in Table S1 in the supplemental material.

10.1128/mSystems.00723-19.6 TABLE S1 List of samples used to construct the metagenomic library. Download Table S1, DOCX file, 0.1 MB. Copyright © 2020 Alexa (Oniciuc) et al. 2020 Alexa (Oniciuc) et al. This content is distributed under the terms of the Creative Commons Attribution 4.0 International license.

### DNA extraction.

For each raw milk sample, 200 ml of raw milk was centrifuged at 5,000 × *g* for 30 min at 4°C. The fat layer was then carefully removed and the supernatant was decanted. Cell pellets were resuspended with a phosphate-buffered saline (PBS) solution, followed by a further centrifugation step under the same conditions. This washing step was repeated once, and finally, cell pellets were resuspended with 1 ml of filter wash buffer solution from the Meta-G-Nome DNA isolation kit (Epicentre Technologies, WI, USA) supplemented with 2 μl of Tween 20. Subsequently, total metagenomic DNA was isolated using the Meta-G-Nome DNA isolation kit according to the manufacturer’s instructions.

For raw milk cheese samples, 10 g of cheese was homogenized with 90 ml of maximum recovery diluent (Merck) using a stomacher. Afterwards, 5 ml of this homogenate was mixed with 45 ml of maximum recovery diluent and then centrifuged at 5,000 × *g* for 30 min at 4°C. Three serial washes with PBS were then performed according to the procedure described for the raw milk samples, followed by the suspension of the pellets with 1 ml of filter wash buffer solution supplemented with 2 μl of Tween 20. Subsequently, total metagenomic DNA was isolated using the Meta-G-Nome DNA isolation kit according to the manufacturer’s instructions.

For processing environment samples, the 40 cotton swabs (Copan Diagnostics) taken at each food business premise were mixed altogether with 100 ml of maximum recovery diluent and incubated under shaking at room temperature for 15 min. The suspension was then filtered through a 0.45-μm filter membrane (Merck), and microorganisms were recovered from the filter with 1 ml of filter wash buffer solution supplemented with 2 μl of Tween 20 by intense vortexing. Subsequently, total metagenomic DNA was isolated using the Meta-G-Nome DNA isolation kit according to the manufacturer’s instructions. The filtration step ensures a higher representation of DNA coming from live intact microbial cells. On the other hand, extracellular DNA, such as that occurring in microbial biofilms, is lost.

For all sample types, DNA was quantified and quality checked by using Qubit (Thermo Fisher Scientific) along with the broad-range DNA quantification assay kit (Thermo Fisher Scientific). The Meta-G-Nome DNA isolation kit was used for all samples as recommended by the Fosmid Library Production kit supplier as a preferred method to isolate inhibitor-free fosmid cloning-ready DNA from unculturable or difficult-to-culture microbial species present in environmental samples.

### Construction of the metagenomic library.

The metagenomic library construction was performed using the Epicentre CopyControl Fosmid Library Production kit (Cambio, Cambridge, England) in strict accordance with the supplier’s instructions. The resulting fragments were cloned into the pCC1FOS vector, harboring a chloramphenicol resistance marker, and transferred into EPI300-T1^R^ E. coli cells. Briefly, size selection was performed on the metagenomic DNA using gel electrophoresis at room temperature overnight at a constant voltage of 35 V. Afterwards, fragments of ∼40 kb were gel extracted from the low-melting-point agarose (Promega, Medical Supply Company, Dublin). Fragments were then ligated with the pCC1FOS vector according to the manufacturer’s instructions and subsequently packaged into the E. coli host cells. Recombinant clones were recovered after plating onto Luria-Bertani agar plates (Difco, Becton, Dickinson & Co., Oxford, England) containing 12.5 μg/ml chloramphenicol (Sigma-Aldrich, St. Louis, MO, USA) (LB-Cm), which were aerobically incubated at 37°C for 48 h. The metagenomic library was stocked in a 96-well format at −80°C.

To confirm the diversity of the metagenomic library, thirty random clones (10 obtained from milk-derived DNA, 10 obtained from cheese-derived DNA, and 10 derived from environmental DNA) were selected. Prior to fosmid DNA isolation, each resistant clone was grown in 100-ml flasks containing LB-Cm broth medium supplemented with 2 μl/ml of CopyControl Fosmid Autoinduction solution (Epicentre) for higher DNA yields, followed by incubation at 37°C for 12 to 16 h and with continuous stirring at 150 rpm. Fosmid DNA was extracted using the FosmidMax DNA purification kit (Epicentre) according to the manufacturer’s instructions and was then subjected to restriction analysis using the ApaI enzyme (Thermo Fisher Scientific) to determine if different DNA insert sequences were present in the metagenomic library. In addition, the inserts of these clones were characterized through partial Sanger sequencing using the primers pCC1-F (5′-GGATGTGCTGCAAGGCGATTAAGTTGG-3′) and pCC1-R (5′-CTCGTATGTTGTGTGGAATTGTGAGC-3′), which target the pCC1FOS vector flanking regions.

### Metagenomic library high-throughput sequencing.

For the sequencing of 9,216 recombinant clones in total, NextSeq libraries were sequenced on the Illumina NextSeq 500, with a NextSeq 500/550 High Output reagent kit v2 (300 cycles), in accordance with the standard Illumina sequencing protocols. Adapter removal and quality trimming of raw metagenomic reads were performed using default parameters of TrimGalore (http://www.bioinformatics.babraham.ac.uk/projects/trim_galore/), a wrapper script for Cutadapt (33) and FastQC (https://www.bioinformatics.babraham.ac.uk/projects/fastqc/). To isolate insert sequences, the high-quality metagenomic reads that mapped to the cloning host genome, vector sequence, and bovine genome were removed using Bowtie2 (34). The resulting SAM files were converted to BAM format and filtered to keep only unmapped paired-end (PE) reads using SAMtools (35). Bedtools (36) was used to convert the remaining reads from BAM to FASTQ format, resulting in 6,565,997 PE reads (68,396 ± 4,554 PE reads per sample). In the absence of a publicly available genome sequence for EPI300-T1^R^, a Bowtie2 database was constructed using the pCC1FOS vector sequence, the genome sequence of E. coli DH10B, and nucleotide sequences of the EZ-Tn*5* DHFR-1 transposon (https://www.lucigen.com/docs/vector/eztn5-dhfr1.txt) (based on personal communication with Lucigen technical support). Taxonomic profiling of the metagenomic bank was performed using Kraken2 (37) with a confidence cutoff of 0.1 and adjusted using Bracken2 (38).

For *in silico* analyses, to identify genes potentially involved in quorum quenching, a custom database built using all available amino acid sequences of known QQ enzymes, as described by LaSarre and Federle (14), was used. These protein names and accession numbers are listed in Table S2. Metagenomic reads were merged using PEAR (39) and then aligned to this database using the “–more-sensitive” preset of the blastx implementation in Diamond (40) and an E value cutoff of 1e−05. When reads aligned to more than one protein in the database, only the hit with the smallest E value was retained. Results are expressed as copies per million reads retained following removal of low-quality, host, vector, and bovine reads. Reads predicted to encode QQ genes were taxonomically classified using Kraken2.

10.1128/mSystems.00723-19.7 TABLE S2 Protein names and accession numbers used for the *in silico* analysis of quorum-quenching determinants. Download Table S2, DOCX file, 0.1 MB. Copyright © 2020 Alexa (Oniciuc) et al. 2020 Alexa (Oniciuc) et al. This content is distributed under the terms of the Creative Commons Attribution 4.0 International license.

10.1128/mSystems.00723-19.8 TABLE S3 List of false discovery rate-corrected *P* values for all pairwise comparisons of alpha diversity between sample sources at phylum and species levels. Download Table S3, DOCX file, 0.1 MB. Copyright © 2020 Alexa (Oniciuc) et al. 2020 Alexa (Oniciuc) et al. This content is distributed under the terms of the Creative Commons Attribution 4.0 International license.

Antimicrobial resistance genes were detected by aligning paired-end metagenomic reads against the MEGARes database, from Lakin and coauthors (41), using the “very-sensitive” preset of Bowtie2. To reduce type I errors, this database was first manually curated to remove any genes corresponding to antimicrobial resistance arising from point mutations. As described above, results are expressed as copies per million reads retained following removal of low-quality, host, vector, and bovine reads, and reads predicted to encode antimicrobial resistance were taxonomically classified using Kraken2.

### Quorum-quenching screening.

To identify clones showing quorum-quenching activity toward autoinducer-1 quorum-sensing molecules, Chromobacterium violaceum DSMZ 30191 was used as a reporter strain. The metagenomic library was grown overnight at 37°C in LB-Cm and then replicated onto LB agar plates using a 96-solid-pin multiblot replicator and incubated overnight at 37°C. After overnight incubation, grown colonies were inactivated through exposure to 99.9% chloroform (Sigma-Aldrich, Ireland) vapor for 30 min and spotted with molten 0.6% LB agar, which was then allowed to solidify at room temperature. The plates were then overlaid with 15 ml 0.3% LB agar inoculated with 150 μl of a C. violaceum overnight culture (obtained in LB broth at 37°C) and incubated for 24 h at 37°C. Afterwards, plates were examined visually for colonies surrounded by a translucent colorless halo.

A similar approach was followed to identify clones showing quorum-quenching activity toward autoinducer-2 quorum-sensing molecules but using Vibrio harveyi DSMZ 19623 as a reporter strain and examining the seeded LB agar plates in an IVIS Lumina II system (PerkinElmer) to identify zones of bioluminescence inhibition, characterized by a lightless halo surrounding candidate clones.

### Antimicrobial resistance screening.

For the antimicrobial resistance functional screening of the metagenomic library, ampicillin was the antibiotic selected as one of the members of the β-lactam antimicrobial class more commonly used in human and animal therapeutics. The MIC of the antibiotic ampicillin for the E. coli host strain was determined in three independent experiments using the agar dilution method. Pools of 96 recombinant clones from the metagenomic library were then recovered through the inoculation of 100 μl of a mixed microbial suspension into a flask containing 50 ml of fresh LB-Cm, which was incubated at 37°C for 24 h on an orbital shaker. Subsequently, 100 μl of the grown culture was surface inoculated onto LB agar plates supplemented with ampicillin at its MIC for the E. coli host strain and incubated at 37°C for 24 h. In parallel, the E. coli host strain was included in the screening approach as a negative control, which showed no growth at all in the presence of the selected concentration of ampicillin. The recombinant clones showing an increased antibiotic resistance were recovered with a sterile loop and stored in the presence of 40% glycerol at −20°C until further use.

### Drug resistance profile of ampicillin-resistant clones.

The susceptibility of the recovered resistant clones to a wide range of antimicrobials was determined by the microdilution method, using a generic Sensititre panel GNX3F- for nonfastidious Gram-negative bacteria (Thermo Fisher Scientific) according to the supplier’s instructions. Susceptibility to 21 antimicrobial agents was tested: amikacin (AMK), doxycycline (DOX), gentamicin (GEN), minocycline (MIN), tobramycin (TOB), tigecycline (TGC), ciprofloxacin (CIP), trimethoprim-sulfamethoxazole (SXT), levofloxacin (LVX), aztreonam (AZT), imipenem (IMI), cefepime (FEP), meropenem (MERO), colistin (COL), polymyxin B (POL), ceftazidime (TAZ), cefotaxime (FOT), ampicillin-sulbactam 2:1 ratio (A/S2), doripenem (DOR), piperacillin-tazobactam (P/T4), and ticarcillin-clavulanic acid (TIM2).

### Molecular characterization of fosmid DNA from selected resistant recombinant clones.

As previously mentioned, for fosmid DNA isolation, clones were grown in 100-ml flasks containing LB-Cm broth medium supplemented with 2 μl/ml of CopyControl Fosmid Autoinduction solution (Epicentre) for higher DNA yields, followed by incubation at 37°C for 12 to 16 h and with continuous stirring at 150 rpm. Fosmid DNA was extracted using the FosmidMax DNA purification kit (Epicentre) according to the manufacturer’s instructions and was then subjected to restriction analysis using ApaI enzyme (Thermo Scientific) to determine if different DNA insert sequences were present. Subsequently, fosmids were sequenced on the Illumina NextSeq 500, with a NextSeq 500/550 High Output reagent kit v2 (300 cycles), in accordance with the standard Illumina sequencing protocols. The KneadData pipeline (v. 0.6.1) (http://huttenhower.sph.harvard.edu/kneaddata) was used to perform quality trimming using Trimmomatic (v. 0.38) (42), and sequences mapping to the cloning host and vector sequences were removed using bmtagger (v. 3.101) (ftp://ftp.ncbi.nlm.nih.gov/pub/agarwala/bmtagger/). The remaining reads were assembled using Unicycler (v. 0.4.7) (43), annotated with Prokka (v. 1.13) (44), and antibiotic resistance was profiled using the Resistance Gene Identifier web portal (45).

### Data availability.

The raw metagenomic sequencing data have been deposited at the European Nucleotides Archive under study accession number PRJEB35062.
