# Supplementary material for: Dairy Products and Dairy-Processing Environments as a Reservoir of Antibiotic Resistance and Quorum-Quenching Determinants as Revealed through Functional Metagenomics
Source: mSystems. 2020 Feb 18;5(1):e00723-19. doi: 10.1128/mSystems.00723-19 (PMC7029220; doi:10.1128/mSystems.00723-19)
Supplement: TABLE S1 [file mSystems.00723-19-st001.docx]

**Supplementary Table S1**. List of samples used to construct the metagenomic library.

| **Environmental samples** | |
| --- | --- |
| Producer 1 (raw milk cow´s cheese) | Forty environmental samples.  At least 15 non food contact environments (drains, floors, walls, trolleys, sinks, etc).  At least 15 food-contact surfaces (cheese vats, milk tanks, molds, etc) |
| Producer 2 (raw milk cow´s cheese) | Forty environmental samples.  At least 15 non food contact environments (drains, floors, walls, trolleys, sinks, etc).  At least 15 food-contact surfaces (cheese vats, milk tanks, molds, etc) |
| Producer 3 (raw milk cow´s cheese) | Forty environmental samples.  At least 15 non food contact environments (drains, floors, walls, trolleys, sinks, etc).  At least 15 food-contact surfaces (cheese vats, milk tanks, molds, etc) |
| Producer 4 (raw milk cow´s cheese) | Forty environmental samples.  At least 15 non food contact environments (drains, floors, walls, trolleys, sinks, etc).  At least 15 food-contact surfaces (cheese vats, milk tanks, molds, etc) |
| **Food samples** | |
| Producer 1 | Semi-hard raw whole cow´s milk cheese, aged for 6 months |
| Producer 2 | Cheddar raw cow´s milk cheese, matured for 6 months |
| Producer 5 | Semi-soft raw cow´s milk cheese, matured for 8 weeks. |
| Producer 6 | Creamy raw milk goat´s cheese, matured for 6 weeks |
| **Milk samples** | |
| Producer 7 | Raw cow´s milk, collected in two different sampling visits |
