# Supplementary material for: Dairy Products and Dairy-Processing Environments as a Reservoir of Antibiotic Resistance and Quorum-Quenching Determinants as Revealed through Functional Metagenomics
Source: mSystems. 2020 Feb 18;5(1):e00723-19. doi: 10.1128/mSystems.00723-19 (PMC7029220; doi:10.1128/mSystems.00723-19)
Supplement: TABLE S2 [file mSystems.00723-19-st002.docx]

**Supplementary Table S2**. Protein names and accession numbers used for the *in silico* analysis of quorum quenching determinants.

| Accession | Protein |
| --- | --- |
| AAD43990.1 | AttM |
| AAG03694.1 | HacB_pseudomonas |
| AAO41113.1 | AiiD |
| AAO47340.1 | AhlK |
| AAP57766.1 | AlhD |
| AAT06802.1 | QsdA |
| AAT68473.1 | AhlM |
| ABU51084.1 | BpiB01 |
| ABU51107.1 | BpiB04 |
| ABU51109.1 | BpiB05 |
| ABU51111.1 | BpiB07 |
| ABV58973.1 | QlcA |
| ADM70923.1 | CarAB |
| AFV15299.1 | QsdH |
| AKI72483.1 | PON1-3 |
| BAB75623.1 | AiiC |
| BAF94155.1 | Aac_MIB015 |
| BAH97082.2 | AiiM |
| BAP32158.1 | AidC |
| CAA75080.1 | Hod |
| EAZ58259.1 | PvdQ |
| gi\|227820442\|ref\|YP_002824413.1\| | DlhR |
| KFN05180.1 | CYP102A1 |
| NGR_b16870 | QsdR1 |
| NP_249723.1 | QuiP |
| NP_396590.1 | AiiB |
| NP_416028.3 | LsrK |
| pdb\|3RKR\|D | BpiB09 |
| pdb\|4G5X\|B | AidH |
| WP_000216581.1 | AiiA |
| WP_011002462.1 | Aac_GMI1000 |
| YP_235052.1 | HacA |
| YP_237923.1 | HacB_B728a |
