# Supplementary material for: Dairy Products and Dairy-Processing Environments as a Reservoir of Antibiotic Resistance and Quorum-Quenching Determinants as Revealed through Functional Metagenomics
Source: mSystems. 2020 Feb 18;5(1):e00723-19. doi: 10.1128/mSystems.00723-19 (PMC7029220; doi:10.1128/mSystems.00723-19)
Supplement: TABLE S3 [file mSystems.00723-19-st003.docx]

**Supplementary Table S3.** List of FDR-corrected *p* values for all pairwise comparisons of alpha diversity between sample sources at phylum and species level.

|  | Phylum | |
| --- | --- | --- |
|  | **Simpson** | **Shannon** |
| Overall | <0.00001 | <0.00001 |
| Environmental vs Environmental + Milk | 0.0001 | 0.00004 |
| Environmental vs Raw Milk | 0.00004 | 0.00004 |
| Environmental vs Raw Milk Cheese 1 | 0.00022 | 0.00172 |
| Environmental vs Raw Milk Cheese 2 | 0.00011 | 0.00011 |
| Environmental + Milk vs Raw Milk | 0.00392 | 0.00281 |
| Environmental + Milk vs Raw Milk Cheese 1 | <0.00001 | <0.00001 |
| Environmental + Milk vs Raw Milk Cheese 2 | <0.00001 | <0.00001 |
| Raw Milk vs Raw Milk Cheese 1 | 0.00002 | 0.00003 |
| Raw Milk vs Raw Milk Cheese 2 | <0.00001 | <0.00001 |
| Raw Milk Cheese 1 vs Raw Milk Cheese 2 | 0.09874 | 0.09039 |
|  | **Species** | |
|  | **Simpson** | **Shannon** |
| Overall | <0.00001 | <0.00001 |
| Environmental vs Environmental + Milk | <0.00001 | <0.00001 |
| Environmental vs Raw Milk | 0.00001 | 0.00001 |
| Environmental vs Raw Milk Cheese 1 | 0.00008 | 0.00008 |
| Environmental vs Raw Milk Cheese 2 | 0.00008 | 0.00002 |
| Environmental + Milk vs Raw Milk | 0.00287 | <0.00001 |
| Environmental + Milk vs Raw Milk Cheese 1 | 0.30407 | 0.34278 |
| Environmental + Milk vs Raw Milk Cheese 2 | 0.00001 | 0.03449 |
| Raw Milk vs Raw Milk Cheese 1 | 0.11377 | 0.05738 |
| Raw Milk vs Raw Milk Cheese 2 | <0.00001 | <0.00001 |
| Raw Milk Cheese 1 vs Raw Milk Cheese 2 | 0.05269 | 0.03449 |
